# Abnormal Eye Position Signals in Interstitial Nucleus of Cajal in Monkeys With “A” Pattern Strabismus

**DOI:** 10.1167/iovs.19-27490

**Published:** 2019-09

**Authors:** Adam Pallus, Michael Mustari, Mark M. G. Walton

**Affiliations:** 1 Washington National Primate Research Center, University of Washington, Seattle, Washington, United States; 2 Department of Ophthalmology, University of Washington, Seattle, Washington, United States; 3 Department of Biological Structure, University of Washington, Seattle, Washington, United States

**Keywords:** exotropia, esotropia, pattern strabismus, interstitial nucleus of Cajal

## Abstract

**Purpose:**

Pattern strabismus is characterized by a cross-axis pattern of horizontal and vertical misalignments. In A-pattern strabismus, for example, a divergent change in the horizontal misalignment occurs on downgaze. Work with nonhuman primate models has provided evidence that this disorder is associated with abnormal cross-talk between brainstem pathways that normally encode horizontal and vertical eye position and velocity. Neurons in the interstitial nucleus of Cajal (INC) are normally sensitive to vertical eye position; in the present study, we test the hypothesis that, in monkeys with pattern strabismus, some INC neurons will show an abnormal sensitivity to horizontal eye position.

**Methods:**

Monkeys were rewarded for fixating a visual target that stepped to various locations on a tangent screen. Single neurons were recorded from INC in one normal monkey, and two with A-pattern strabismus. Multiple linear regression analysis was used to estimate the preferred direction for each neuron.

**Results:**

In the normal monkey, all INC neurons had preferred directions within 20° of pure vertical (either up or down). The preferred directions were significantly more variable in the monkeys with pattern strabismus, with a minority being more sensitive to horizontal eye position than vertical eye position. In addition, the vertical eye position sensitivity was significantly less in the monkeys with strabismus.

**Conclusions:**

In pattern strabismus, neurons in INC show neurophysiological abnormalities consistent with a failure to develop normal tuning properties. Results were consistent with the hypothesis that, in pattern strabismus, INC receives an abnormally strong signal related to horizontal eye position.

Strabismus is a common disorder, characterized by a chronic inability to simultaneously orient both eyes toward an object of interest. For a subset of patients, the horizontal and vertical misalignments vary with eye position along the orthogonal axis.^1^ When the horizontal and vertical eye positions are plotted simultaneously in these patients, the misalignments form a pattern that often resembles the letters “A” or “V.”^2^–^6^ Thus, this condition is referred to as “pattern strabismus.”

Numerous studies employing nonhuman primate models of the infantile strabismus syndrome have shown that, when binocular vision is disturbed for a prolonged period in infancy, both visual and oculomotor areas of the brain develop abnormally.^7^,^8^ Most of these monkeys develop at least a mild “A” or “V” pattern, and some show a strong pattern strabismus.^3^,^5^,^6^ In human patients, pattern strabismus is typically described, and treated, as overaction or underaction of the oblique muscles.^1^,^9^ However, evidence from nonhuman primate studies has shown convincingly that this is, at best, simplistic. For example, single unit recordings from oculomotor nucleus have shown that modulation of the firing rates of superior rectus, inferior rectus, and medial rectus motoneurons could account for most of the contextually inappropriate cross-axis movement of the ipsilateral eye when the contralateral eye pursues a visual target moving along the orthogonal axis.^4^,^6^ Indeed, we^10^–^12^ and other groups^5^,^13^,^14^ have suggested that pattern strabismus has a more complex, neurological origin involving abnormal cross-talk between brainstem areas that normally would encode signals related to either the horizontal or vertical component of eye movements. At present very little is known about where in the brainstem this might occur but, since both saccades and smooth pursuit show similar patterns of directional disconjugacy,^2^–^4^,^6^,^14^ it is reasonable to hypothesize that the cross-talk is occurring at the level of structures that are shared by multiple oculomotor subsystems. One such structure is the interstitial nucleus of Cajal (INC), which contains neurons that carry signals related to vertical eye position during fixation of static targets, smooth pursuit, and the vestibulo-ocular reflex (VOR).^15^–^18^ Some of these neurons also show high frequency bursts of spikes associated with the vertical component of saccades and also encode, through their tonic firing rates, the vertical eye position during the intersaccadic periods.^17^,^18^ Importantly, one neuroanatomical study has shown that projections exist from a structure believed to play a crucial role in the integration of horizontal eye velocity signals, the nucleus prepositus hypoglossi (NPH), to INC in normal monkeys.^19^ This suggests a possible route by which signals related to horizontal eye position might reach the vertical eye position pathway.

In a recent paper, we proposed three control systems models to account for the directional disconjugacies of saccades and the cross-axis misalignments of pattern strabismus.^8^ A key assumption of these models is that abnormal cross-talk affects the two eyes differently. In “A” pattern strabismus, for example, the divergent change in horizontal strabismus angle on down-gaze could occur if INC neurons that drive inferior rectus motoneurons and superior oblique motoneurons serving the right and left eyes receive *different* horizontal eye position signals. On the basis of these considerations, therefore, we hypothesized that a subset of INC neurons would show an abnormal sensitivity to horizontal eye position in monkeys with pattern strabismus and that their preferred directions would differ for the two eyes.

## Methods

### Subjects and Surgical Procedures

Data were collected from three macaque monkeys, including one with normal eye alignment (monkey N1), one with “A” pattern exotropia (monkey XT1), and one with “A” pattern esotropia (monkey ET1). N1 and ET1 were both *Macaca mulatta* and Monkey XT1 was *Macaca nemestrina*. For monkey ET1, a chronic esotropia was experimentally induced in infancy by injecting botulinum toxin into the lateral rectus muscle during the first week of life.^20^,^21^ This animal also received three follow-up injections between the ages of 4 months and 2 years. At the time that recording experiments began (>3 years of age), the animal had esotropia, with a horizontal misalignment that typically ranged from 10° to 25°. Monkey XT1 underwent a bilateral medial rectus tenotomy^22^ during the first week of life, which resulted in a chronic exotropia with a horizontal misalignment that typically ranged from 15° to 35°.

All surgical and experimental procedures were approved by the Institutional Animal Care and Use Committee at University of Washington, and were in full compliance with the National Institutes of Health Guide for the Care and Use of Laboratory Animals and the ARVO Statement for the Use of Animals in Ophthalmic and Vision Research. In order to prevent movement of the head during recording sessions, a titanium head post (Crist Instruments Co., Inc., Hagerstown, MD, USA) was attached to the skull using titanium bolts. A recording chamber was positioned over a 16-mm craniotomy, at a location chosen to maximize the chances of an electrode track at the center of the chamber passing through INC. The positions of both eyes were measured with the magnetic search coil technique, using eye coils that were chronically implanted beneath the conjunctiva.^23^,^24^ Additional details regarding our surgical procedures are available in previously published studies.^25^,^26^

### Behavioral Tasks and Visual Display

Monkeys were rewarded with a small amount of applesauce, every 0.3 to 0.5 seconds, for maintaining fixation on a 0.25° red laser spot. This visual target was back-projected onto a flat screen that was positioned 57 cm from the eyes. The target stepped to a new location every 1.5 to 5 seconds, which encouraged the monkey to make saccades to continue receiving the reward. Target locations were under computer control, chosen randomly from a set of possible horizontal and vertical Cartesian coordinates (0°, 2°, 4°, 6°, 8°, 10°, 12°, 15°, 18°, 22° left, right, up, or down). Since the strabismic monkeys were unable to simultaneously direct both eyes to the target, the reward was delivered if at least one eye was directed within 5° of the target. A sinusoidal smooth pursuit task was used to obtain Hess plots for the monkeys with strabismus. For this task, the target moved horizontally or vertically, at a frequency of 0.1 to 0.2 Hz. This was done only to obtain a visual representation of the pattern strabismus and so the data were not analyzed further.

Monkey ET1 consistently became anxious and refused to work when one eye was covered. This behavior did not improve, even after weeks of attempting to acclimate the animal to a pair of shutter goggles or an opaque eye patch. However, under binocular viewing conditions, the animal voluntarily switched the viewing eye periodically. Monkey XT1 had large angle exotropia, which is known to be associated with significant suppression of visual information originating from the temporal retinae.^27^,^28^ Indeed, we have consistently observed that, when one eye is patched, monkeys with large-angle exotropia have difficulty seeing targets presented in the hemifield contralateral to the viewing eye. For this reason, in our previous studies, we have avoided patching either eye in these animals.^3^,^10^,^11^,^29^,^30^ However, targets presented on the right side of the screen are consistently viewed with the right eye and targets presented on the left side are consistently viewed with the left eye. When the exotropia is large, this means that the nonviewing eye will be 25° to 40° away from the target, viewing darkness. Thus, although we did not collect data under monocular viewing conditions, we were able to obtain sufficient data, for some recordings, to compare the preferred directions for right-eye-on-target and left-eye-on-target conditions.

### Unit Recording and Localization of INC

Extracellular recordings were made from individual neurons in INC, using tungsten and glass microelectrodes (Frederick-Haer, Bowdoin, ME, USA). Initial localization of INC was based on the presence of tonic and burst-tonic activity that modulated with vertical eye position^31^ and the proximity of the recording sites to other, easily identifiable, structures such as oculomotor nucleus, the supraoculomotor area, and the rostral interstitial nucleus of the medial longitudinal fasciculus.

As noted earlier in this article, INC is believed to function as a vertical neural integrator. For that reason, the eyes remain at, or near, their new locations following the offset of a microelectrical stimulation train.^32^ In contrast, microstimulation of oculomotor nucleus bypasses neural integration and, therefore, the eyes quickly return to their starting positions with an exponential time course after the end of each train of pulses.^32^ Thus, for additional confirmation that our recordings were made from INC, we used microstimulation (100 ms, 300–400 Hz, 20 μA) to verify that the final eye position was maintained after the offset of each train.

### Data Analysis

Target presentation, reward, and all other aspects of the experiment were controlled using custom scripts, running in Spike 2 (Cambridge Electronic Design, Cambridge, UK). This same software package was also used for preliminary assessment of each recording. Data were then exported into Matlab (MathWorks, Natick, MA, USA) for more detailed analysis. Horizontal and vertical eye velocity and acceleration were estimated using seven-point parabolic differentiation.

Saccades were identified using the same algorithm we have used in several recent studies that involved monkeys with strabismus.^3^,^11^ Briefly, movement onset was defined as the time that vectorial eye velocity first crossed a threshold of 50°/s. The algorithm marked saccade offset when either of two conditions was met: (1) vectorial eye velocity dropped below 50°/s or (2) vectorial eye velocity fell below 100°/s and the absolute value of acceleration dropped below 10,000°/s^2^. The more complex criteria were used for movement offset to ensure that abnormally large postsaccadic drifts (which sometimes resulted in a re-acceleration of the eye) were not erroneously included as part of the saccade.^11^

For the strabismic animals, the horizontal and vertical misalignments are mathematically (but not physiologically) equivalent to horizontal and vertical vergence angle in a normal animal and were therefore computed using equation 1:

\newcommand{\bialpha}{\boldsymbol{\alpha}}\newcommand{\bibeta}{\boldsymbol{\beta}}\newcommand{\bigamma}{\boldsymbol{\gamma}}\newcommand{\bidelta}{\boldsymbol{\delta}}\newcommand{\bivarepsilon}{\boldsymbol{\varepsilon}}\newcommand{\bizeta}{\boldsymbol{\zeta}}\newcommand{\bieta}{\boldsymbol{\eta}}\newcommand{\bitheta}{\boldsymbol{\theta}}\newcommand{\biiota}{\boldsymbol{\iota}}\newcommand{\bikappa}{\boldsymbol{\kappa}}\newcommand{\bilambda}{\boldsymbol{\lambda}}\newcommand{\bimu}{\boldsymbol{\mu}}\newcommand{\binu}{\boldsymbol{\nu}}\newcommand{\bixi}{\boldsymbol{\xi}}\newcommand{\biomicron}{\boldsymbol{\micron}}\newcommand{\bipi}{\boldsymbol{\pi}}\newcommand{\birho}{\boldsymbol{\rho}}\newcommand{\bisigma}{\boldsymbol{\sigma}}\newcommand{\bitau}{\boldsymbol{\tau}}\newcommand{\biupsilon}{\boldsymbol{\upsilon}}\newcommand{\biphi}{\boldsymbol{\phi}}\newcommand{\bichi}{\boldsymbol{\chi}}\newcommand{\bipsi}{\boldsymbol{\psi}}\newcommand{\biomega}{\boldsymbol{\omega}}\begin{equation}\tag{1}S = {P_{left}} - {P_{right}} \end{equation}

where *S* is the strabismus angle (either horizontal or vertical), *P_left_* is the horizontal (or vertical) position of the left eye, and *P_right_* is the horizontal (or vertical) position of the right eye.

Spike 2 software was used for preliminary evaluation of unit isolation, but the final spike times were based on a custom algorithm that we have used in previous studies.^11^,^29^ This algorithm initially detects candidate spikes based on a simple voltage threshold, but it then rejects spikes with amplitudes that differ from those of recent spikes by more than a user-selectable percentage (usually set at 30%), or those that occur at implausibly short interspike intervals. In our testing, this algorithm has proven to be ideal for tonically active neurons with spike amplitudes that vary over time. In these cases, the algorithm is generally able to reject smaller spikes from background units.

Some INC neurons show saccade-related bursts in addition to eye position related activity (burst-tonic), while others (tonic neurons) do not.^32^,^33^ To distinguish between the two groups without making a priori assumptions about direction preference, we compared the mean firing rate during the saccade to that during the time interval between 300 and 400 ms after saccade offset. We then computed a burst index (BI), using the same equation used for that purpose in our recent study of near response cells in strabismus (equation 2).^34^

\newcommand{\bialpha}{\boldsymbol{\alpha}}\newcommand{\bibeta}{\boldsymbol{\beta}}\newcommand{\bigamma}{\boldsymbol{\gamma}}\newcommand{\bidelta}{\boldsymbol{\delta}}\newcommand{\bivarepsilon}{\boldsymbol{\varepsilon}}\newcommand{\bizeta}{\boldsymbol{\zeta}}\newcommand{\bieta}{\boldsymbol{\eta}}\newcommand{\bitheta}{\boldsymbol{\theta}}\newcommand{\biiota}{\boldsymbol{\iota}}\newcommand{\bikappa}{\boldsymbol{\kappa}}\newcommand{\bilambda}{\boldsymbol{\lambda}}\newcommand{\bimu}{\boldsymbol{\mu}}\newcommand{\binu}{\boldsymbol{\nu}}\newcommand{\bixi}{\boldsymbol{\xi}}\newcommand{\biomicron}{\boldsymbol{\micron}}\newcommand{\bipi}{\boldsymbol{\pi}}\newcommand{\birho}{\boldsymbol{\rho}}\newcommand{\bisigma}{\boldsymbol{\sigma}}\newcommand{\bitau}{\boldsymbol{\tau}}\newcommand{\biupsilon}{\boldsymbol{\upsilon}}\newcommand{\biphi}{\boldsymbol{\phi}}\newcommand{\bichi}{\boldsymbol{\chi}}\newcommand{\bipsi}{\boldsymbol{\psi}}\newcommand{\biomega}{\boldsymbol{\omega}}\begin{equation}\tag{2}BI = {{{{\overline {FR} }_{Sac}} - {{\overline {FR} }_{Post}}} \over {{{\overline {FR} }_{Sac}} + {{\overline {FR} }_{Post}}}} \end{equation}

$\newcommand{\bialpha}{\boldsymbol{\alpha}}\newcommand{\bibeta}{\boldsymbol{\beta}}\newcommand{\bigamma}{\boldsymbol{\gamma}}\newcommand{\bidelta}{\boldsymbol{\delta}}\newcommand{\bivarepsilon}{\boldsymbol{\varepsilon}}\newcommand{\bizeta}{\boldsymbol{\zeta}}\newcommand{\bieta}{\boldsymbol{\eta}}\newcommand{\bitheta}{\boldsymbol{\theta}}\newcommand{\biiota}{\boldsymbol{\iota}}\newcommand{\bikappa}{\boldsymbol{\kappa}}\newcommand{\bilambda}{\boldsymbol{\lambda}}\newcommand{\bimu}{\boldsymbol{\mu}}\newcommand{\binu}{\boldsymbol{\nu}}\newcommand{\bixi}{\boldsymbol{\xi}}\newcommand{\biomicron}{\boldsymbol{\micron}}\newcommand{\bipi}{\boldsymbol{\pi}}\newcommand{\birho}{\boldsymbol{\rho}}\newcommand{\bisigma}{\boldsymbol{\sigma}}\newcommand{\bitau}{\boldsymbol{\tau}}\newcommand{\biupsilon}{\boldsymbol{\upsilon}}\newcommand{\biphi}{\boldsymbol{\phi}}\newcommand{\bichi}{\boldsymbol{\chi}}\newcommand{\bipsi}{\boldsymbol{\psi}}\newcommand{\biomega}{\boldsymbol{\omega}}{\overline {FR} _{Sac}}$ represents the mean firing rate during the saccade. A positive value indicates a higher firing rate during the saccade than in the postsaccadic period. A negative value would indicate that the firing rate was lower during the saccade. A neuron was classified as burst-tonic if the mean value of *BI* exceeded 0.25 for saccades larger than 10° in any direction.

For each neuron, estimation of the horizontal and vertical eye position sensitivities were based on measuring the mean firing rates during periods of steady fixation. A period of steady fixation was identified when all of the following criteria were met: (1) No saccades were detected during the preceding 100 ms, (2) vectorial eye velocity remained below 25°/s, and (3) the duration of the fixation was at least 500 ms. All fixation periods ended 50 ms before the next detected saccade. The 25°/s criteria was used because monkey ET1 sometimes showed a nystagmus (quick phases were down-right).

The mean firing rate was measured during each identified period of steady fixation. The resulting data were then fit with equation 3:

\newcommand{\bialpha}{\boldsymbol{\alpha}}\newcommand{\bibeta}{\boldsymbol{\beta}}\newcommand{\bigamma}{\boldsymbol{\gamma}}\newcommand{\bidelta}{\boldsymbol{\delta}}\newcommand{\bivarepsilon}{\boldsymbol{\varepsilon}}\newcommand{\bizeta}{\boldsymbol{\zeta}}\newcommand{\bieta}{\boldsymbol{\eta}}\newcommand{\bitheta}{\boldsymbol{\theta}}\newcommand{\biiota}{\boldsymbol{\iota}}\newcommand{\bikappa}{\boldsymbol{\kappa}}\newcommand{\bilambda}{\boldsymbol{\lambda}}\newcommand{\bimu}{\boldsymbol{\mu}}\newcommand{\binu}{\boldsymbol{\nu}}\newcommand{\bixi}{\boldsymbol{\xi}}\newcommand{\biomicron}{\boldsymbol{\micron}}\newcommand{\bipi}{\boldsymbol{\pi}}\newcommand{\birho}{\boldsymbol{\rho}}\newcommand{\bisigma}{\boldsymbol{\sigma}}\newcommand{\bitau}{\boldsymbol{\tau}}\newcommand{\biupsilon}{\boldsymbol{\upsilon}}\newcommand{\biphi}{\boldsymbol{\phi}}\newcommand{\bichi}{\boldsymbol{\chi}}\newcommand{\bipsi}{\boldsymbol{\psi}}\newcommand{\biomega}{\boldsymbol{\omega}}\begin{equation}\tag{3}FR(t - {t_d}) = a + {k_{hor}}H + {k_{vert}}V \end{equation}

where *H* and *V* are the horizontal and vertical eye positions, respectively, and *K_hor_* and *K_vert_* represent the estimated sensitivities to horizontal and vertical eye position, respectively. The expression (*t* − *t_d_*) is a time shift to compensate for neural processing delays. It was set to 20 ms for all recordings, but note that, since the firing rate was averaged over fixation periods that typically lasted for at least several hundred milliseconds, the effect of this time shift was minimal. Equation 3 was used to perform separate fits for the two eyes. The model fits were performed using Matlab's curve fit tool, which provides 95% confidence bounds for all parameter estimates. The horizontal and vertical eye position sensitivities were considered statistically significant if the 95% confidence bound did not include zero.

The next step was to estimate a preferred direction for each neuron. To do this, the sensitivities to horizontal and vertical eye position, derived from equation 2, were treated as Cartesian coordinates and then converted to polar coordinates using the “cart2pol” function in Matlab. For example, for a neuron with equal sensitivities to rightward and upward eye position, this procedure would yield a preferred direction of 45°. The preferred direction was estimated separately for the right and left eyes. Equation 4 was used to estimate the absolute deviation of the preferred direction from pure vertical, for each eye and for each neuron:

\newcommand{\bialpha}{\boldsymbol{\alpha}}\newcommand{\bibeta}{\boldsymbol{\beta}}\newcommand{\bigamma}{\boldsymbol{\gamma}}\newcommand{\bidelta}{\boldsymbol{\delta}}\newcommand{\bivarepsilon}{\boldsymbol{\varepsilon}}\newcommand{\bizeta}{\boldsymbol{\zeta}}\newcommand{\bieta}{\boldsymbol{\eta}}\newcommand{\bitheta}{\boldsymbol{\theta}}\newcommand{\biiota}{\boldsymbol{\iota}}\newcommand{\bikappa}{\boldsymbol{\kappa}}\newcommand{\bilambda}{\boldsymbol{\lambda}}\newcommand{\bimu}{\boldsymbol{\mu}}\newcommand{\binu}{\boldsymbol{\nu}}\newcommand{\bixi}{\boldsymbol{\xi}}\newcommand{\biomicron}{\boldsymbol{\micron}}\newcommand{\bipi}{\boldsymbol{\pi}}\newcommand{\birho}{\boldsymbol{\rho}}\newcommand{\bisigma}{\boldsymbol{\sigma}}\newcommand{\bitau}{\boldsymbol{\tau}}\newcommand{\biupsilon}{\boldsymbol{\upsilon}}\newcommand{\biphi}{\boldsymbol{\phi}}\newcommand{\bichi}{\boldsymbol{\chi}}\newcommand{\bipsi}{\boldsymbol{\psi}}\newcommand{\biomega}{\boldsymbol{\omega}}\begin{equation}\tag{4}AbsDe{v_V} = \left| {{D_{ideal}} - {D_{Actual}}} \right| \end{equation}

where *AbsDev_V_* is the absolute deviation of the preferred direction from vertical, *D_Actual_* is the estimated preferred direction for the neuron, and *D_ideal_* is the nearest “pure” vertical direction (90° or 270°, whichever is closer to *D_Actual_*).

## Results

**Figure 1:**
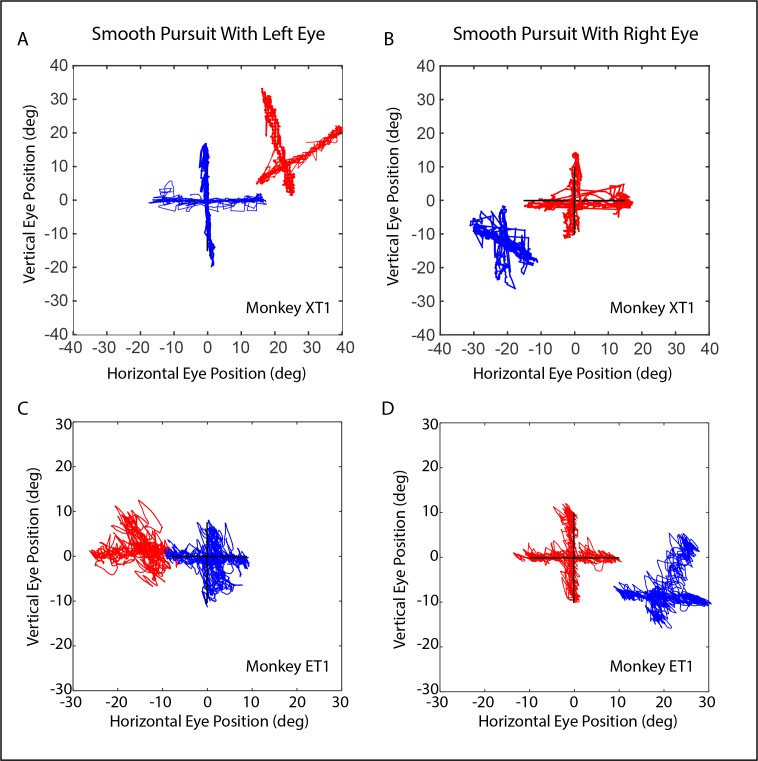
Hess plots, based on horizontal and vertical sinusoidal smooth pursuit with the left eye (A, C) and the right eye (B, D). (Panels C and D re-plot the same data depicted in figure 2 of Walton and Mustari.^12^) Left eye position is shown in blue; right eye position is shown in red. Data from exotropic monkey XT1 are shown in the top row (A, B), and data from exotropic monkey ET1 are shown in the bottom row (C, D). Both monkeys showed a robust A-pattern, such that a divergent change in the horizontal strabismus angle occurs on downgaze.

Figure 1 shows Hess plots for monkeys XT1 and ET1, obtained during horizontal and vertical smooth pursuit. Both monkeys were able to successfully pursue the moving target with either eye but, when the viewing eye followed a target moving horizontally or vertically, the fellow eye usually moved at an oblique angle. For monkey XT1, this effect was very slight during vertical pursuit with the right eye (panel B) but was quite robust during vertical pursuit with the left eye (panel A).

Under binocular viewing conditions both monkeys voluntarily switched the fixating eye. Monkey ET1 exhibited a clear preference for the right eye, while monkey XT1 typically used the left eye to fixate targets to the left of straight ahead and the right eye to fixate targets presented to the right of straight ahead.

As noted in Methods, microstimulation was used to verify that the electrode was in INC.

We recorded 26 neurons from monkey N1 that showed statistically significant sensitivity to eye position. Of these, 19 were classified as tonic and seven as burst-tonic. For the monkeys with strabismus, we recorded 69 neurons with significant eye position sensitivity (28 tonic and 41 burst-tonic).

### Rate-Position Curves

**Figure 2:**
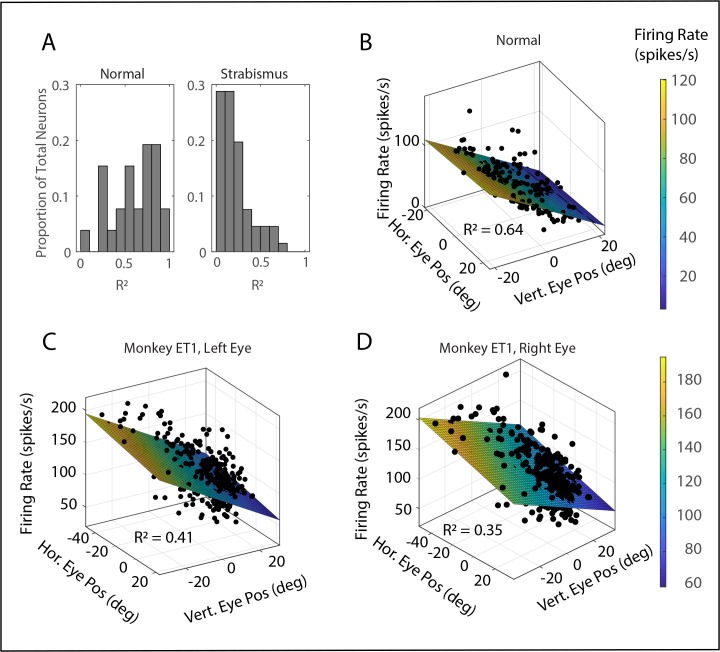
Model fits of equation 3, which uses horizontal and vertical eye position to predict the tonic firing rate during periods of steady fixation (see Methods). The color of the plane represents the predicted firing rate. (A) R^2^ values for all neurons that showed a statistically significant relationship between horizontal and/or vertical eye position and firing rate. For monkey N1, there was a broad distribution, but a clear majority had R^2^ values > 0.5. In the strabismic monkeys, by contrast, one can see a large peak below 0.2, and there were very few with R^2^ values > 0.5. (B) Example fit for a typical neuron (i.e., slopes and R^2^ values close to the mean for this animal) recorded from monkey N1. (C, D) Model fits for the left eye (C) and right eye (D) for a typical example neuron recorded from monkey ET1. Note the relatively poor R^2^ values (which were still slightly higher than the mean values for the strabismic monkeys) and the sensitivity to horizontal eye position (slight for the left eye and robust for the right eye).

**Figure 3:**
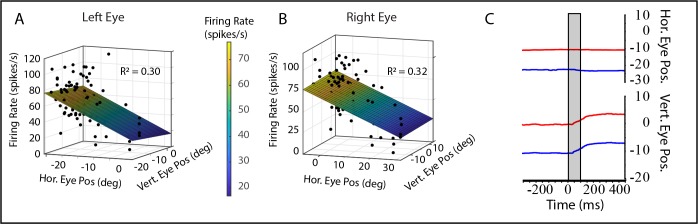
Model fits for a tonic neuron recorded from monkey XT1 for the left eye (A) and right eye (B), with a firing rate that was primarily related to horizontal eye position. Conventions for panels A and B are the same as those used for Figure 2B–D. (C) Example microstimulation (20 μA, 400 Hz, 100 ms) of the site at which the neuron in panels A and B was recorded. Red = position of the right eye; blue = position of the left eye. The gray shaded area indicates the period of microstimulation. Note that the eyes remain at the new locations after the end of the stimulation train, which indicates that the artificially imposed signal was integrated. This does not happen when oculomotor nucleus is stimulated.

Figure 2 shows the results of fitting the data with equation 3. The neuron in panel B was recorded from monkey N1 and was selected to be as typical as possible for that animal (i.e., the absolute values of horizontal and vertical eye position sensitivities and the *R*^2^ value were all close to the mean). Panels C and D show the fits for the left and right eyes, respectively, for one neuron recorded from monkey ET1. This cell also showed horizontal and vertical eye position sensitivities that were close to the mean values, averaged across all recordings from strabismic animals. The *R*^2^ values for this cell, for both eyes, were somewhat higher than the means across all strabismic animals (left eye: 0.41 for this cell, 0.28 across all cells; right eye: 0.35 for this cell, 0.28 across all cells). Both neurons are clearly sensitive to vertical eye position, but the goodness of fit is substantially better for the neuron recorded from the normal animal. Panel A shows that this was typical across our data set; for the normal monkey, *R*^2^ values > 0.5 were obtained for the majority of neurons. For the strabismic animals, however, the goodness of fit was < 0.2 for the majority of neurons in our sample. A two-tailed *t*-test showed that the mean *R*^2^ value was significantly larger for monkey N1 (*P* < 0.01). A close examination of panels C and D of Figure 2 reveals another abnormality: the neuron recorded from monkey ET1 shows a small sensitivity to horizontal eye position. This effect is strongest for the right eye (D), but it was statistically significant for both eyes (i.e., the 95% confidence interval for the slopes did not include zero). Figure 3 shows the model fit for another example tonic neuron, recorded from monkey ET1. The firing rate is clearly related to horizontal eye position, and there is little or no sensitivity to vertical eye position.

**Figure 4:**
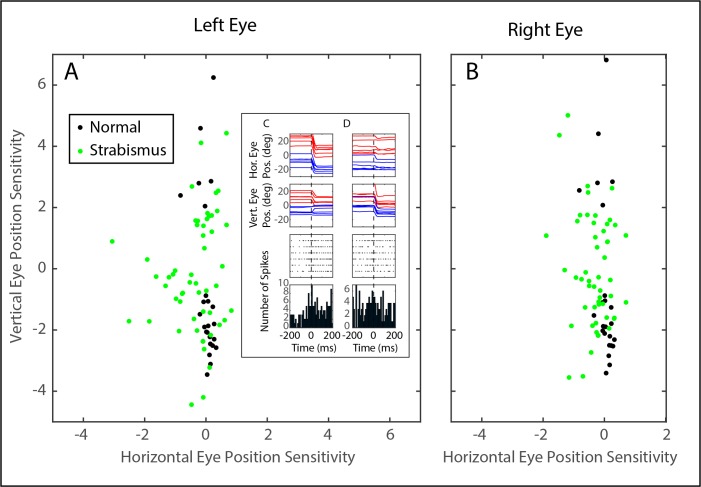
Comparison of estimated vertical and horizontal eye position sensitivities for the left (A) and right (B) eyes for all neurons for which model fits yielded R^2^ values of at least 0.1. For monkey N1 (black), only one of the neurons in our sample had a vertical eye position sensitivity between −1 and 1. For the strabismic monkeys (green), the absolute value of the vertical sensitivity was less than one for 32 of the 50 neurons with R^2^ values ≥ 0.1. In addition, 8 of 50 neurons recorded from strabismic monkeys had estimated horizontal eye position sensitivities < −1 for the left eye. This was the case for 6 of 50 neurons for the right eye. Overall, therefore, INC neurons recorded from monkeys with pattern strabismus tended to have weaker sensitivities to vertical eye position and stronger sensitivities to horizontal eye position. Inset: Raster plots for an example neuron, recorded from monkey XT1, showing a consistent increase in the tonic firing rate for leftward saccades (C). Due to directional saccade disconjugacy,^3^,^14^ many of these saccades also had a downward component for the right eye, but no consistent modulation was observed for downward saccades that occurred in the absence of a large leftward component (D).

Figure 2A shows that the goodness of fit was quite poor for some of the neurons in our sample, particularly for the monkeys with strabismus. Including such neurons in analyses of preferred directions could be misleading. With that in mind, we excluded any neuron with an *R*^2^ below 0.1. This resulted in the exclusion of 1 of 26 neurons from monkey N1, 4 of 26 from monkey ET1, and 15 of 43 from monkey XT1. Interestingly, 10 of 25 (40%) of the neurons recorded from monkey N1 showed a statistically significant sensitivity to horizontal eye position. This included four of seven of the burst-tonic neurons and 8 of 18 of the tonic neurons. For the monkeys with strabismus, 31 of 50 (62%) showed a statistically significant sensitivity to horizontal eye position for the left eye, and 35 of 50 (70%) showed a significant sensitivity to horizontal eye position for the right eye. For the left eye, this included 17 of 41 of the burst-tonic neurons and 13 of 28 of the tonic neurons. For the right eye, this included 19 of 41 of the burst-tonic neurons and 11 of 28 of the tonic neurons. The mean absolute value of the horizontal position sensitivity was significantly larger for both eyes in the monkeys with pattern strabismus (left eye = 0.51; right eye = 0.41) than for monkey N1 (0.17) (two-tailed *t*-test, *P* < 0.01 for both comparisons). The mean absolute value of the vertical position sensitivity, however, was significantly lower for both eyes in the monkeys with pattern strabismus (left eye = 1.29; right eye = 1.21) than for monkey N1 (2.33) (two-tailed *t*-test, *P* < 0.001 for both comparisons). Figure 4 compares the estimated horizontal and vertical eye position sensitivities for the left (A) and right eyes (B) for all neurons for which the model fits yielded *R*^2^ values of at least 0.1. The vertical eye position sensitivities tended to be weaker for the strabismic monkeys than for the normal animal. The horizontal eye position sensitivities, however, tended to be stronger for the monkeys with strabismus; this was particularly true for the left eye. Figure 4C shows an example of microstimulation of a site in left INC of monkey XT1. Vertical movement was observed for both eyes and, importantly, the eyes remained near their new locations after the end of microstimulation.

**Figure 5:**
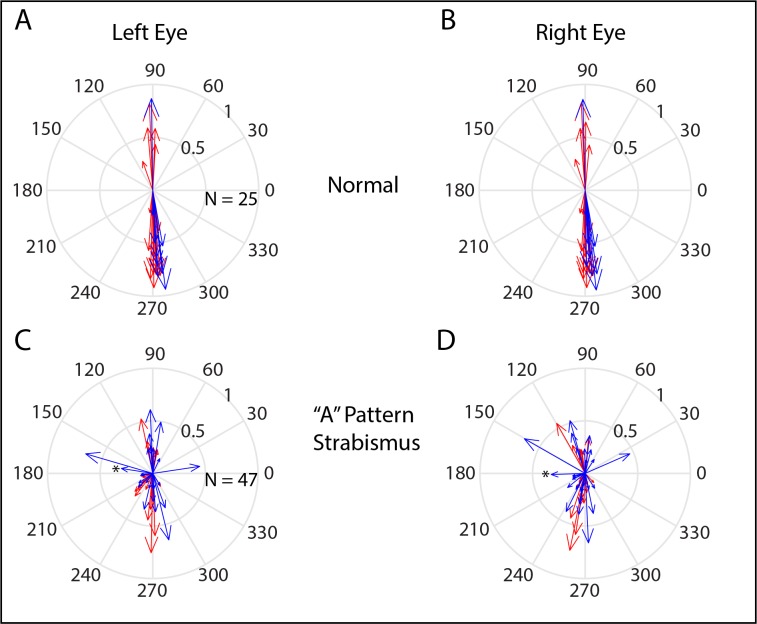
Preferred directions of INC neurons. The direction of each arrow represents the estimated preferred direction for one neuron. Arrow lengths indicate that R^2^ values obtained from fitting the data with equation 3. Neurons recorded from INC on the right side of the brain are shown in red; neurons recorded from left INC are shown in blue. (A, B) The estimated preferred directions of neurons recorded from monkey N1 were always within 20° of vertical. (C, D) The preferred directions of neurons recorded from the strabismic animals were much more variable; a subset were more sensitive to horizontal eye position than vertical eye position. The asterisk at the point of one of the arrows identifies the example neuron shown in Figure 3.

**Figure 6:**
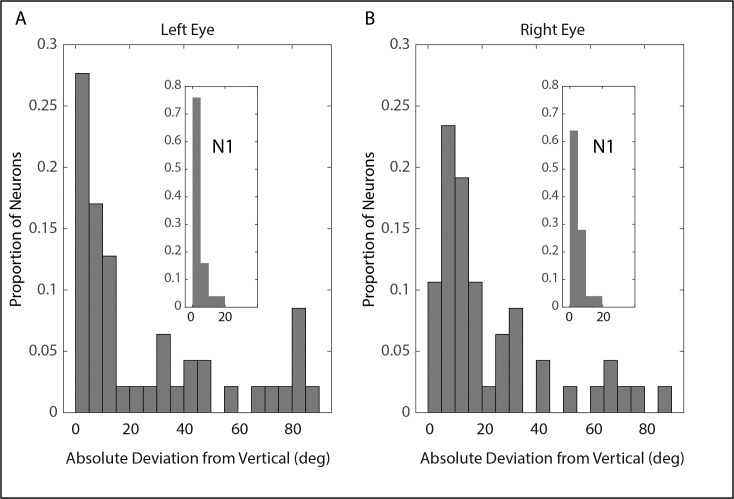
Distributions of absolute deviations of estimated preferred directions from pure vertical for the left (A) and right (B) eyes for the monkeys with strabismus. Insets show the corresponding distributions for monkey N1.

Figure 5 shows the preferred directions for all neurons for which the model fits yielded *R*^2^ values of at least 0.1, estimated using the procedure described in Methods. Because the majority of neurons in monkeys XT1 and ET1 had reasonably normal preferred directions, and because we were reluctant to make comparisons between *n* = 1 esotrope and *n* = 1 exotrope, we elected to pool data from the two monkeys with strabismus (panels C and D). The length of each arrow corresponds to the *R*^2^ value of the fit obtained from equation 3. Note that all 25 of the neurons recorded from monkey N1 had preferred directions within 20° of vertical (either upward or downward). This was also true for the majority of the neurons recorded from monkeys ET1 and XT1. For a minority of neurons recorded from these strabismic monkeys, however, the absolute value of the horizontal sensitivity was greater than the absolute value of the vertical sensitivity. This was the case for 12 of 47 neurons for the left eye and 7 of 47 for the right eye. For six neurons, the horizontal sensitivity was greater than the vertical for both eyes. The mean absolute deviation from vertical was significantly larger for the strabismic animals, compared with monkey N1 (left eye: N1 = 4.22°, strabismus = 26.47°, two-tailed *t*-test *P* < 0.001; right eye: N1 = 4.45°, strabismus = 23.65°, two-tailed *t*-test *P* < 0.001). Figure 6 shows the distributions of AbsDev_V_ for each eye for the strabismic monkeys. Insets show the corresponding distributions for monkey N1. It is clear from Figure 6 that there were many neurons with abnormal preferred directions in the strabismic animals, but a close examination of panels C and D of Figure 5 shows that the majority of these neurons had *R*^2^ values < 0.25 (but note that there were several exceptions to this).

For both saccade^3^ and smooth pursuit^2^ tasks, directional errors are typically observed for the nonviewing eye in pattern strabismus. This being the case, one might wonder whether the preferred directions of INC neurons change depending on which eye the subject uses to fixate the visual target. Unfortunately, this proved to be a difficult analysis to perform because of two issues; first, neither animal performed the task well when one eye was patched (see Methods) and second, the low *R*^2^ values for our model fits for many of the neurons in our sample made it difficult to obtain robust estimates of preferred direction when the data were limited to fixations with a particular eye. With these limitations in mind, we estimated the preferred directions separately for right-eye-on-target and left-eye-on-target conditions only when the following criteria were met: (1) the *R*^2^ values exceeded 0.2 for both eyes and (2) at least 20 fixations were detected for both fixating-eye conditions. Twelve neurons satisfied these criteria. When the mean preferred directions were compared for the right-eye-on-target and left-eye-on-target conditions, no significant differences were found for either eye (two-tailed *t*-tests: left eye, *P* = 0.82; right eye, *P* = 0.91).

## Discussion

In this study, we examined the tuning properties of eye-position related neurons in INC and found two major abnormalities. First, compared with the normal control, our monkeys with A-pattern strabismus showed an abnormally broad distribution of preferred directions. Secondly, the multiple linear regression analysis (equation 2) yielded much lower *R*^2^ values in the strabismic monkeys.

We have previously reported similar effects for saccade-related burst neurons in paramedian pontine reticular formation (PPRF); in the normal monkey the preferred directions were always within 30° of horizontal, but in two monkeys with A-pattern strabismus, we found an abnormally broad distribution of preferred directions on one side of the brain.^11^ In the present study, too, the most abnormal preferred directions (i.e., those that were mostly horizontal for both eyes) were found in left INC (Figs. 5C, 5D). The significance of this is unclear, but it is worth noting that the two monkeys used in the present study were not the same ones used in our PPRF study. This raises the possibility that disturbed directional tuning on one side of the brain may be a common feature of brainstem oculomotor regions in pattern strabismus.

Overall, the multiple linear regression analysis (equation 3) yielded remarkably poor *R*^2^ values for the neurons recorded from the monkeys with A-pattern strabismus. It is possible that the neurons in our sample with the lowest *R*^2^ values primarily encoded something other than vertical eye position. For example, some INC neurons carry signals related to ocular torsion,^32^ which we were not able to measure. However, we consider it unlikely that this could fully account for the poor model fits. For example, in Figure 2A one can see that, for the majority of neurons recorded from the normal animal, the model yielded *R*^2^ values > 0.5. By contrast, the paucity of good model fits in the strabismic monkeys is striking. Moreover, the low *R*^2^ values are consistent with previous studies that have shown disruption of normal tuning across many brain areas in monkeys with strabismus, including reductions in the number of binocularly responsive neurons and disparity sensitivity in V1,^35^–^37^ middle temporal cortex (MT),^21^ and medial superior temporal cortex (MST),^38^ and poor correlations between the number of spikes in saccade-related bursts and horizontal amplitude in PPRF.^11^ Similarly, although neurons in the supraoculomotor area are sensitive to horizontal strabismus angle (mathematically equivalent to vergence angle) in monkeys with experimentally induced strabismus, the sensitivity and *R*^2^ values are notably reduced compared with the vergence position sensitivity in normal monkeys.^13^,^34^ Nonetheless, we cannot entirely exclude the possibility that the low *R*^2^ values for the model fits in the strabismic monkeys reflects, in part, an abnormality in the distributions of cell types within INC.

Abnormal ocular torsion is common in pattern strabismus; indeed this is one reason why ophthalmologists frequently suspect involvement of the oblique muscles.^1^,^8^ However, it is clear that torsional abnormalities alone cannot account for the cross-axis patterns of misalignments in this disorder. First, torsional abnormalities are not always observed in human patients with pattern strabismus.^39^ Second, during horizontal and vertical smooth pursuit, in monkeys with pattern strabismus, both horizontal and vertical rectus muscle motoneurons serving the nonviewing eye modulate their discharge in association with the inappropriate cross-axis movement.^4^,^6^ In this situation, therefore, an inappropriate signal is sent to the horizontal or vertical rectus motoneurons of one eye and not the other. Third, conjugate horizontal eye movements are evoked by microstimulation of some sites in PPRF of monkeys with pattern strabismus, but microstimulation of other sites in the same animal evokes directionally disconjugate movements with a strong vertical component.^10^

It is believed that some INC neurons carry signals related to ocular torsion; microstimulation of right INC evokes clockwise rotation while stimulation of left INC evoked counter-clockwise rotation.^32^ If this structure receives an abnormal drive from the horizontal pathway in pattern strabismus, this would provide a parsimonious explanation for torsional abnormalities in this disorder.

Synaptic connectivity undergoes considerable development and experience-dependent pruning during early postnatal life.^40^–^42^ With these observations in mind, it is likely that prolonged disruption of normal binocular vision during this sensitive period leads to a cascade of abnormalities that results in permanent disruptions of normal tuning across many visual and oculomotor areas.^7^,^8^ In the present study, the paucity of rate-position curves with high *R*^2^ values in the monkeys with strabismus is particularly striking since INC directly drives vertically acting motoneurons and is, therefore, quite close to the motor output.

In a recent report, we considered three computational models to account for the cross-axis directional disconjugacy that characterizes pattern strabismus.^12^ Two of the models assumed abnormal cross-talk between NPH and INC, such that a subset of eye-position sensitive neurons in the latter structure would be expected to display an abnormal sensitivity to horizontal eye position. The results of the present study are consistent with this prediction. However, the issue is somewhat more complex than that. As was pointed out in the discussion section of our earlier report, any attempt to model pattern strabismus must reconcile directional disconjugacy of saccades^3^,^14^ with the fact that both humans and monkeys with pattern strabismus are able to make accurate saccades with either eye fixating.^43^,^44^ The fundamental problem is that cross-talk at such a late stage of processing introduces a directional error to at least one eye, with few distal brain regions remaining to correct it. We proposed that the cerebellum switches between different adaptive states, depending on which eye is to be directed to the target. Since saccade adaptation is conjugate in strabismus,^45^ the fundamental directional disconjugacy would be largely preserved, even though the subject is able to correctly bring either eye to the target. This cerebellar-derived correction would change the direction of the requested movement (i.e., the inputs to downstream structures like the neural integrators), but the cross-talk itself would, presumably, be unaffected. Thus, the models we proposed in our earlier study do not necessarily predict that the preferred directions of INC neurons would differ, depending on which eye views the target.
